# Thick filament mechano-sensing is a calcium-independent regulatory mechanism in skeletal muscle

**DOI:** 10.1038/ncomms13281

**Published:** 2016-10-31

**Authors:** L. Fusi, E. Brunello, Z. Yan, M. Irving

**Affiliations:** 1 Randall Division of Cell and Molecular Biophysics and BHF Centre of Research Excellence, King's College London, London SE1 1UL UK

## Abstract

Recent X-ray diffraction studies on actively contracting fibres from skeletal muscle showed that the number of myosin motors available to interact with actin-containing thin filaments is controlled by the stress in the myosin-containing thick filaments. Those results suggested that thick filament mechano-sensing might constitute a novel regulatory mechanism in striated muscles that acts independently of the well-known thin filament-mediated calcium signalling pathway. Here we test that hypothesis using probes attached to the myosin regulatory light chain in demembranated muscle fibres. We show that both the extent and kinetics of thick filament activation depend on thick filament stress but are independent of intracellular calcium concentration in the physiological range. These results establish direct control of myosin motors by thick filament mechano-sensing as a general regulatory mechanism in skeletal muscle that is independent of the canonical calcium signalling pathway.

## Abstract (web summary)

Recent data suggest that muscle contraction is regulated by thick filament mechano-sensing in addition to the well-known thin filament-mediated calcium signalling pathway. Here the authors provide direct evidence that myosin activation in skeletal muscle is controlled by thick filament stress independently of calcium.

Contraction of skeletal and heart muscle is triggered by a transient increase in intracellular free calcium ion concentration. The calcium ions bind to troponin in the actin-containing thin filaments, initiating a change in the structure of the thin filaments that permits myosin heads or motor domains from the interdigitating thick filaments to bind to actin and generate force or filament sliding^1^. However electron microscopy studies have shown that the myosin molecule is switched OFF by an asymmetric arrangement of the two heads of the myosin molecule folded back against the myosin tail, which inhibits their ATP-ase activity^2^,^3^. This arrangement is conserved across a remarkably wide range of muscle types and species, suggesting the existence of a general mechanism in which the myosin motors are made unavailable for actin binding in resting muscle because they are sequestered in helical tracks on the surface of the thick filaments^4^,^5^,^6^,^7^,^8^. This sequestered, ‘super-relaxed' or ‘OFF' state of the thick filament also inhibits the ATP-ase activity of resting skeletal muscle^9^,^10^. Exit from the structural and functional OFF state is controlled by phosphorylation of the myosin regulatory light chain in vertebrate smooth muscle^2^,^11^,^12^, and by calcium binding to myosin in some invertebrate striated muscles^13^. In vertebrate skeletal muscle, recent X-ray diffraction studies on isolated single cells led to the proposal of a novel signalling pathway controlling release of the myosin motors from the OFF state: that the thick filament is switched ON by mechanical stress^14^. According to this proposal, a small number of constitutively ON myosin motors sense the regulatory state of the thin filament. When the load on the muscle is high, the force generated by the constitutively ON motors produces a stress in the thick filament that triggers the release of the remaining motors. When the external load is low, the constitutively ON motors are sufficient to drive rapid muscle shortening, and the thick filament remains predominantly OFF^14^. In this concept, thick filament regulation acts like an automatic gearbox for muscle contraction, efficiently coupling myosin function to the external load on the muscle.

Here we test and further develop this concept of the thick filament as a regulatory mechano-sensor and elucidate its relationship to the well-known thin filament-mediated calcium signalling pathway. The X-ray measurements described above mainly used intact muscle fibres activated by electrical stimulation; here we use demembranated muscle fibres, in which the intracellular calcium concentration and the thick filament stress can be independently controlled. We imposed thick filament stress in the relaxed (low-calcium) state by exploiting the fact that titin links between the tips of the thick filaments and the ends of the sarcomere transmit the passive stress generated at long sarcomere length to the thick filaments^15^,^16^,^17^. We extended this protocol to activating calcium concentrations by inhibiting active force with a specific myosin inhibitor. We determined the kinetics of the OFF/ON transition in the thick filament on the millisecond timescale using force-clamp technology. In all these protocols, we used polarized fluorescence from bifunctional rhodamine probes to simultaneously determine the orientation of part of the myosin motor—the C-lobe of the myosin regulatory light chain (RLC)—in the muscle fibre as a precise, reproducible and time-resolved measure of the structural OFF/ON transition in the thick filament^7^.

The results presented below show that the amplitude and speed of thick filament activation depend on thick filament stress but are independent of calcium concentration in the physiological range, and establish thick filament mechano-sensing as a general second regulatory mechanism in skeletal muscle that is independent of the well-known calcium signalling pathway.

## Results

### RLC orientation is sensitive to passive stress

**Figure 1:**
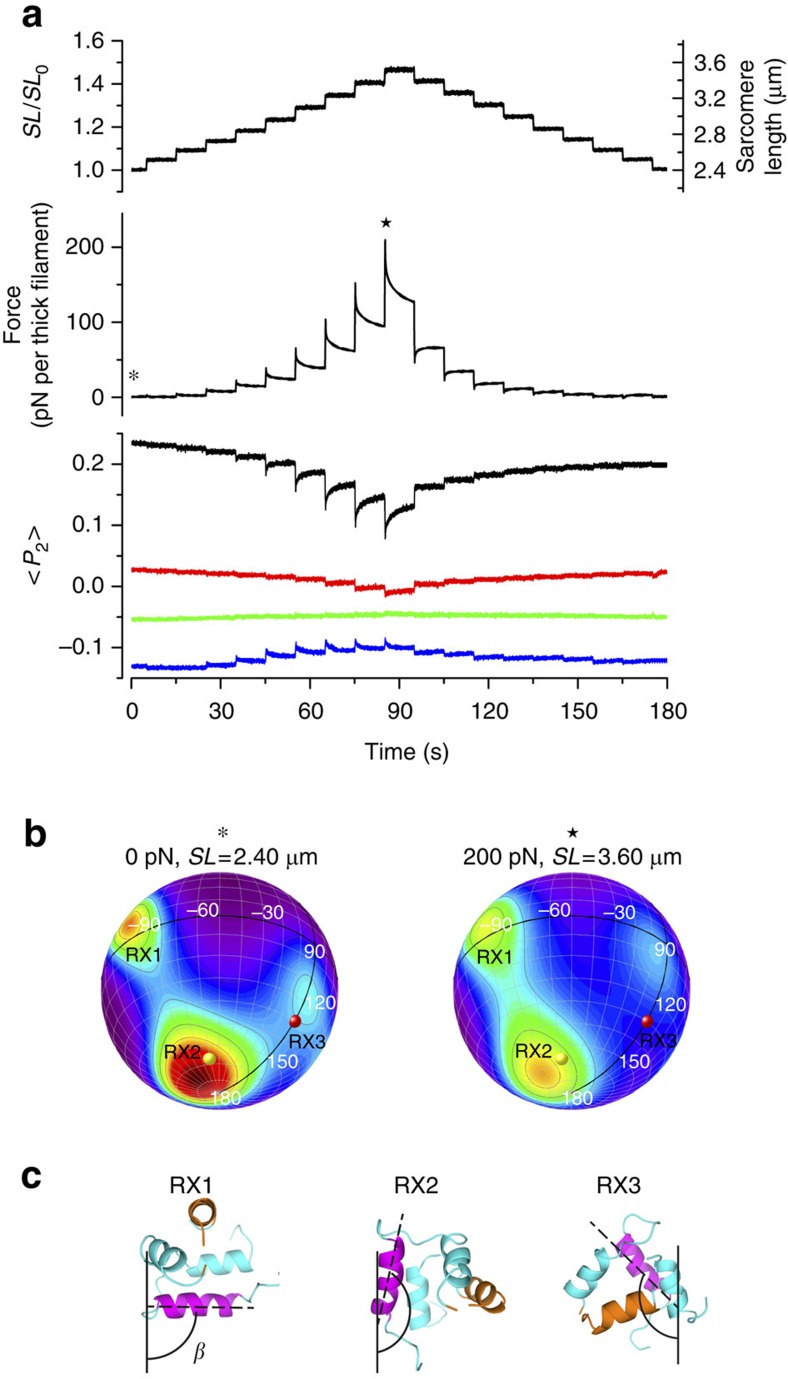
Stress-induced changes in the orientation of the RLC-C-lobe in relaxed muscle. (**a**) Sarcomere length change, estimated from total fluorescence intensity (see Supplementary Methods and Supplementary Fig. 1) (*SL*_0_=2.40 μm), passive force and <*P*_2_> for four RLC probes^7^ (E-probe, black; FG-probe, red; H-probe, green; G-probe, blue) recorded during a staircase of stretches and releases applied to a relaxed muscle fibre at 25 °C in the presence of 5% Dextran. (**b**) Maximum entropy (ME) spherical contour plots of the probability distribution of RLC C-lobe orientations at 0 (asterisk) and 200 pN (star; see text for details); red and yellow spheres denote the orientations determined by electron microscopy of isolated thick filaments (3DTP^6^). (**c**) Graphical representations of RLC C-lobe orientations RX1-3 from **b** with respect to the filament axis (black vertical line), with E and G helices in magenta and orange, respectively (see also Supplementary Fig. 6 of Fusi *et al*.^7^ for comparison with RLC orientations in 3DTP).

When a demembranated single fibre from rabbit psoas muscle is stretched in relaxing (low-calcium) conditions from a sarcomere length of 2.40 μm, a typical *in vivo* value in which there is maximal overlap between the interdigitating thick and thin filaments, to 3.60 μm, where the overlap between thick and thin filaments is greatly reduced, the passive stress in the fibre increases to about ∼80 kPa (∼1/3 of the maximum calcium-activated force), corresponding to a force of about 200 pN per thick filament (Fig. 1a). In this sarcomere length range the passive force is predominantly due to the reversible extension of titin links between the ends of the thick filaments and the ends of the sarcomere^15^,^16^,^17^. To provide an overview of the sarcomere length-dependence and kinetics of this phenomenon, we applied a series of ramp stretches of ∼5% fibre length (*L*_0_) and 0.5 s duration at intervals of 10 s, followed by a series of ramp releases to regain the original fibre length (Fig. 1a, upper panel). Thick-filament force increases roughly exponentially with increasing sarcomere length in this protocol (Fig. 1a, middle panel; Supplementary Figs 1,2 and 3a), but the increase during each stretch partially decays in the 10 s interval before the next stretch, and the force response to a 5% release is characteristically different from that to a stretch in the same sarcomere length range.

This staircase passive stretch-release protocol was imposed on a series of demembranated muscle fibres in which the native myosin RLC had been partially replaced by an RLC labelled at one of four locations on the surface of its C-lobe by a bifunctional rhodamine probe, as described previously^7^. The orientation of each probe in the fibre was measured continuously by polarized fluorescence and expressed as the order parameter <*P*_2_> (Fig. 1a, lower panel), which is a measure of how parallel the probe dipole is to the filament axis^7^,^18^. The measurements were made at 25 °C, pCa 9 in the presence of 5% Dextran, conditions in which the OFF state of the thick filament is strongly favoured at sarcomere length 2.40 μm (ref. 7). The distinct values of <*P*_2_> in those conditions for the four probes, on the E-helix (black), G-helix (blue), H-helix (green) and cross-linking the F and G helices (red) of the RLC C-lobe reflect its preferred orientation in the OFF state of the thick filament^7^.

Increasing the sarcomere length in the staircase protocol decreased <*P*_2_> for the E-helix probe (black) in a manner that mirrored the changes in passive force (Fig. 1a and Supplementary Fig. 3a). *<P*_2_> did not change significantly for stretches up to ∼2.7 μm sarcomere length but decreased at longer sarcomere lengths as passive force increased. During the same staircase protocol <*P*_2_> for the FG-helix probe (red) decreased, <*P*_2_> for the G-helix probe (blue) increased, whereas <*P*_2_> for the H-helix probe (green) did not change significantly. The time course of the <*P*_2_> response to each stretch for each probe was also similar to that of the passive force response; the <*P*_2_> change during each stretch was larger at longer sarcomere length, partially reversed during the subsequent visco-elastic recovery between stretches, and showed a similar asymmetry between the stretch and release staircases. Moreover the change in <*P*_2_>, like that in passive tension, was maintained for several hundreds of seconds after a ramp stretch, until the length change was reversed (Supplementary Fig. 4). Plots of <*P*_2_> against force during the stretch/release protocol were roughly linear during the two staircases (Supplementary Figs 3b and 5), with some hysteresis between stretch and release. These results show that the orientation of the C-lobe of the RLC is directly related to the passive force and, since the latter is predominantly due to the titin links at the ends of the thick filaments, to the thick filament stress, as expected from the mechano-sensing hypothesis.

Because the angular co-ordinates of the four bifunctional probes in the RLC structure are known^7^,^19^, the measured <*P*_2_> and <*P*_4_> values for the four probes (Supplementary Fig. 5) can be used to calculate the orientation of the C-lobe of the RLC with respect to the thick filament axis^20^. More precisely, the method determines the smoothest C-lobe orientation distribution that is consistent with the eight measured order parameters. This maximum entropy distribution is then plotted as a contour map on the surface of a sphere (Fig. 1b), where β (latitude) denotes the angle between the RLC E-helix and the filament axis (Fig. 1c), and γ (longitude) denotes the rotation of the RLC around the E-helix^7^.

The maximum entropy distribution of the RLC C-lobe at 2.40 μm sarcomere length and zero passive stress (Fig. 1b, left) is similar to that described previously^7^. There are three preferred orientations: an equatorial lobe (RX1) at *β*=90° and *γ*=−90°, a polar lobe (RX2) at *β*=165°and *γ*=−70° and a minor lobe (RX3) at *β*=120°, *γ*=0°. The conformation of the C-lobe in each of these three orientations with respect to a vertical thick filament axis is shown graphically in Fig. 1c. The RX2 and RX3 orientations are similar to those in the ‘free' and ‘blocked' myosin motors respectively in the so-called interacting heads motif observed in three-dimensional reconstructions of isolated thick filaments^6^,^21^ (Fig. 1b; yellow and red spheres, respectively). About 65% of myosin motors in the muscle fibre are in the RX2 or RX3 orientations at zero stress^7^.

At 3.60 μm sarcomere length, with a passive force of about 200 pN per thick filament corresponding to about 30% of the active isometric force in these conditions, the RX2 peak becomes weaker and the RX3 peak moves towards the equator (Fig. 1b, right). Detailed quantification of the fraction of RLCs in each peak depends on the choice of integration limits, but roughly 15% of the myosin motors move from the more polar RX2 and RX3 orientations to equatorial orientations, that is, from parallel to perpendicular orientations with respect to the filament axis. Since the E-helix of the RLC is roughly parallel to the lever arm of the myosin motor, we conclude that the lever arms of these myosin motors also move to more perpendicular orientations at higher filament stress.

### Kinetics of RLC orientation changes in response to stress

**Figure 2:**
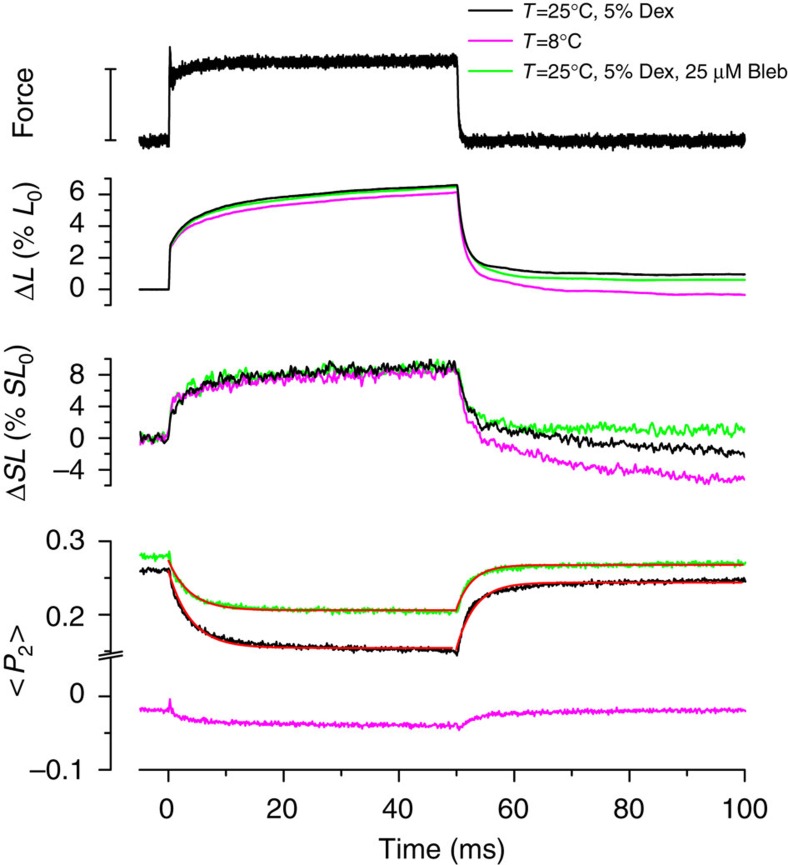
Orientation changes of the E-helix probe in response to a force step in relaxed muscle. Upper panel: force (bar length=110 pN per thick filament); middle panels: fibre length change (Δ*L*) relative to initial fibre length (*L*_0_) and sarcomere length change (Δ*SL*) relative to initial sarcomere length (*SL*_0_=2.8 μm); lower panel: <*P*_2_> for the E-helix probe. Black: 25 °C, 5% Dextran; green: 25 °C, 5% Dextran, 25 μM Blebbistatin; magenta: 8 °C, no Dextran or Blebbistatin. Red: single-exponential fits following force increase (black: 256 s^−1^, green: 270 s^−1^) and decrease (black: 313 s^−1^, green: 367 s^−1^).

The stretches in the staircase protocol of Fig. 1 are relatively slow, and do not allow the kinetics of changes in myosin motor orientation to be resolved on the physiological timescale of muscle activation^14^. We therefore used a rapid force-step/clamp protocol to determine the time course of the change in RLC orientation. Relaxed muscle fibres were stretched slowly to 2.8 μm sarcomere length, at which passive force is very low in the steady state. They were then stretched in 0.2 ms to increase passive force to 110 pN per thick filament. Force was clamped at that value by feedback control for 50 ms, then decreased to the original low value in 0.5 ms (Fig. 2, upper trace). Fibre length (Fig. 2, Δ*L*, black) increased by about 2% *L*_0_ during the 0.26 ms force step (phase 1), and by a further 4% *L*_0_ during the subsequent 50 ms of visco-elastic extension at constant force (phase 2). Sarcomere length (Fig. 2, Δ*SL*, black) estimated from fluorescence intensity as described in Supplementary Methods (Supplementary Fig. 1) had a similar biphasic time course, increasing by about 2% *SL*_0_ in phase 1 and a further 6% *SL*_0_ in phase 2.

The orientation of the RLC C-lobe reported by <*P*_2_> for the E-helix probe did not change during phase 1 of this protocol (Fig. 2, black); Δ<*P*_2_> in phase 1 was —0.002±0.002 (mean±s.d., *n*=4 fibres). <*P*_2_> did decrease during phase 2, by 0.10±0.01 (mean±s.d., *n*=4 fibres), with an exponential time course corresponding to a rate constant of ∼250 s^−1^ (Fig. 2, red). When the passive force was rapidly removed, <*P*_2_> recovered to its initial value with an exponential time course corresponding to a rate constant of ∼300 s^−1^. The changes in <*P*_4_> had similar kinetics (Supplementary Fig. 6). These results show that the change in RLC orientation induced by a change in passive stress is not elastic and does not accompany the instantaneous component of the change in sarcomere length, but is associated with an intrinsic rate constant of 250–300 s^−1^ in the conditions in Fig. 2.

### Stress-sensing by the RLC requires an OFF thick filament

The results described in the previous section (Fig. 2, black) were obtained at 25 °C, pCa 9 in the presence of 5% Dextran, conditions in which the thick filament is predominantly OFF^7^. In those conditions passive stretch induces a transition towards the ON, motors-perpendicular, state (Fig. 1b). To test whether the response of the RLC to passive stress depends on the initial OFF/ON state of the thick filament, we repeated the force-clamp protocol at 8 °C in the absence of Dextran, conditions which favour the ON state^7^. The mechanical response of the fibre to a force step in these conditions (Fig. 2 and Supplementary Fig. 6, magenta) was similar to that at 25 °C, 5% Dextran (black), but the <*P*_2_> response was greatly reduced (Δ<*P*_2_>=−0.020±0.007, mean±s.d., *n*=4 fibres). These results show that the change in RLC orientation produced by passive stress requires the OFF state of the thick filament.

To further test this conclusion, we added 25 μM Blebbistatin, which stabilizes the OFF state of the thick filament^7^,^22^, to the relaxing solution at 25 °C in the presence of 5% Dextran (Fig. 2, green). Blebbistatin does not alter the mechanical response of the fibre to a step increase in stress (Fig. 2, Δ*L*, Δ*SL* green), but slightly increases <*P*_2_> for the E-helix probe in the absence of stress^7^, corresponding to a slightly more OFF thick filament. Imposition of the 110 pN-force step decreased <*P*_2_> for the E-helix probe by 0.07±0.01 (mean±s.d., *n*=4 fibres) with a rate constant of ∼270 s^−1^, similar to the response observed in the absence of Blebbistatin.

### Stress-sensing by the RLC is independent of [Ca^2+^]

**Figure 3:**
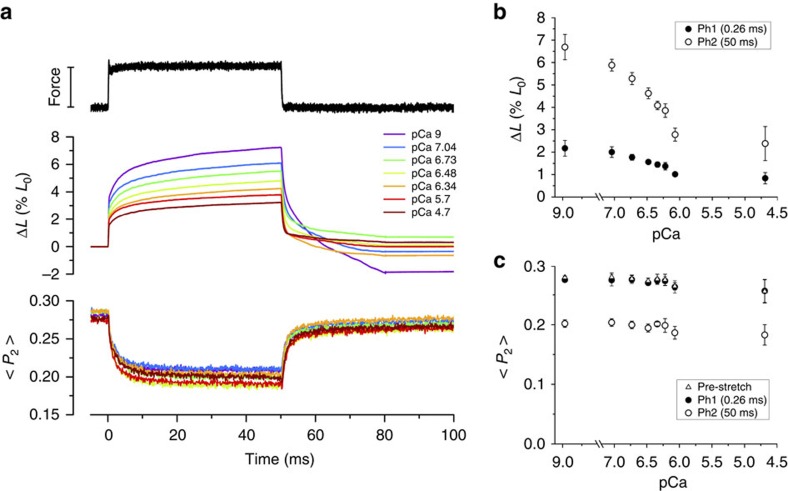
Calcium-dependence of the response to a force step in the presence of Blebbistatin. (**a**) Force (bar length=120 pN per thick filament), fibre length change (Δ*L*) and <*P*_2_> for the E-helix probe at different calcium concentrations. (**b**) Calcium-dependence of Δ*L* at 0.26 ms (phase 1, filled circles) and 50 ms (phase 2, open circles) (mean±s.d., *n*=3 fibres). (**c**) <*P*_2_> for the E-helix probe before the force step (triangles), at the end of phase 1 (filled circles) and phase 2 (empty circles; mean±s.d., *n*=3 fibres). Initial sarcomere length 2.80 μm, 25 °C, 5% Dextran, 25 μM Blebbistatin.

The finding that the stress-induced changes in RLC orientation are essentially unaltered in the presence of 25 μM Blebbistatin (Fig. 2) allowed us to investigate how those changes depend on [Ca^2+^] in conditions in which the active force of a muscle fibre is almost completely inhibited^23^. For this purpose we used a force clamp protocol similar to that in Fig. 2; Blebbistatin was always present but [Ca^2+^] was varied from the relaxed value (ca 1 nM) to a value (20 μM) that would produce full activation in the absence of Blebbistatin (Fig. 3).

The increase in fibre length produced by a force step to 120 pN per thick filament in these conditions was smaller at higher [Ca^2+^], both at the end of phase 1 (Fig. 3b, filled circles) and of phase 2 (Fig. 3b, open circles). Fibre lengthening in both phases was ∼2.5 times smaller at pCa 4.7 ([Ca^2+^]=20 μM) than at pCa 9 ([Ca^2+^]=1 nM), indicating that the passive compliance (=Δ*L*/Δ*F*) of the fibre is lower at higher [Ca^2+^]. A similar Ca^2+^-dependent decrease in half-sarcomere compliance, from ∼600 to ∼200 nm hs^−1^ MPa^−1^, was calculated from the sarcomere length changes (Supplementary Figs 7a and 8). The latter value, measured at pCa 4.7, is similar to a recent estimate of the compliance in parallel with the myosin motors during active isometric contraction^24^. The decrease in the passive compliance of the half-sarcomere at higher [Ca^2+^] is likely to be associated with a decrease of that of the PEVK region of titin^25^,^26^,^27^,^28^. However at higher [Ca^2+^], the small fraction of force-generating myosin motors that are not inhibited by 25 μM Blebbistatin and responsible for the ca 6% residual force under these conditions could also contribute to the reduction in half-sarcomere compliance.

<*P*_2_> for the E-helix probe was almost independent of pCa at low stress in the presence of Blebbistatin (Fig. 3a,c, triangles). The small decrease at pCa 4.7 (Δ<*P*_2_>=−0.02±0.02, mean±s.d., *n*=3 fibres), about 7% of the change observed in the absence of Blebbistatin (Δ<*P*_2_>= −0.26±0.02, mean±s.d., *n*=4 fibres), is consistent with the residual ∼6% force remaining in its presence, indicating that there is no direct effect of [Ca^2+^] on the orientation of the C-lobe of the RLC. Moreover, the decrease in <*P*_2_> induced by a 120-pN force step was also almost independent of [Ca^2+^], both at the end of phase 1 (Fig. 3c, filled circles) and phase 2 (Fig. 3c, open circles). <*P*_2_> decreased by 0.076±0.002 (mean±s.d., *n*=3 fibres) with a rate constant of ∼300 s^−1^ independent of [Ca^2+^], despite the [Ca^2+^]-dependence of the increase in fibre length and sarcomere length in these conditions.

These results show that the change in the orientation of the RLC and its kinetics depend on the passive stress in the thick filament but are independent of [Ca^2+^] and of the change in sarcomere length or half-sarcomere compliance.

### Dependence of RLC orientation on thick filament stress

**Figure 4:**
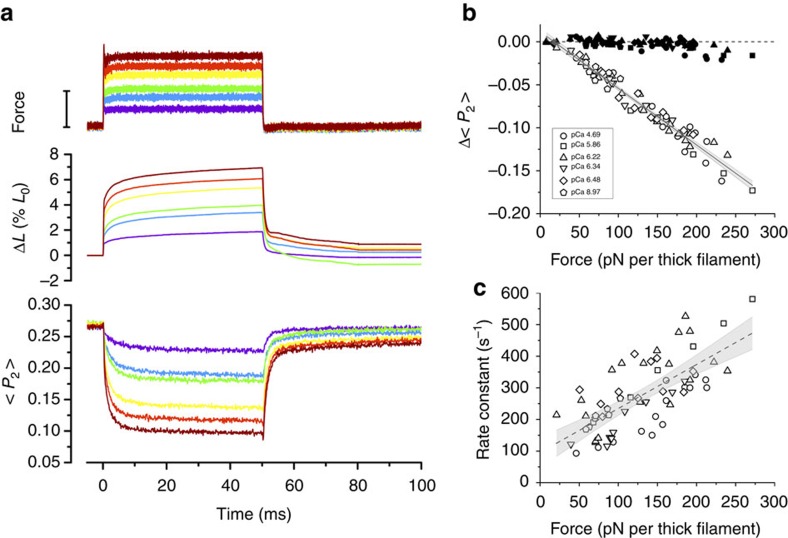
Thick-filament stress controls the size and speed of the RLC orientation change. (**a**) Force (bar length=120 pN per thick filament), fibre length change (Δ*L*) and <*P*_2_> for the E-helix probe at pCa=5.86 for different sizes of force step. (**b**) Force-dependence of the change in <*P*_2_> (Δ<*P*_2_>) at the end of phase 1 (filled symbols) and phase 2 (empty symbols; pooled data from five fibres). Solid line: linear regression of phase 2 data with 95% confidence band shown in grey: slope=(−6.6±0.2)·10^−4^ pN^−1^, intercept=0.012±0.002. (**c**) Rate constant of the <*P*_2_> change in response to the force increase (symbols as in **b**). Dashed line: linear regression with 95% confidence band in grey; slope=1.4±0.2 s^−1^ pN^−1^, intercept=96±24 s^−1^. Initial sarcomere length 2.80 μm, 25 °C, 5% Dextran, 25 μM Blebbistatin.

To characterize the stress-dependence of the amplitude and kinetics of the change in RLC orientation, we applied force pulses in the range ∼20–300 pN per thick filament at pCa between 9 and 4.7 in the presence of Blebbistatin (Fig. 4a and Supplementary Fig. 7b). The change in <*P*_2_> during phase 1 was independent of force over most of the range studied, but <*P*_2_>decreased slightly at forces >200 pN per thick filament (Fig. 4b, filled symbols). The change in <*P*_2_> measured at the end of phase 2 decreased in proportion to the size of the force step, independently of pCa (Fig. 4b, open symbols). The rate constant of the <*P*_2_> transient elicited by a stretch was force-dependent, increasing from ∼100 to ∼500 s^−1^ as the force was increased from 20 to 300 pN per thick filament (Fig. 4c). When force was returned to zero after 50 ms, <*P*_2_> recovered its initial value with a rate constant of about 400 s^−1^ (Supplementary Fig. 9). These results indicate that the orientation of the C-lobe of the RLC and the kinetics of changes in its orientation are controlled by thick filament stress.

### Changes in RLC orientation during active contraction

**Figure 5:**
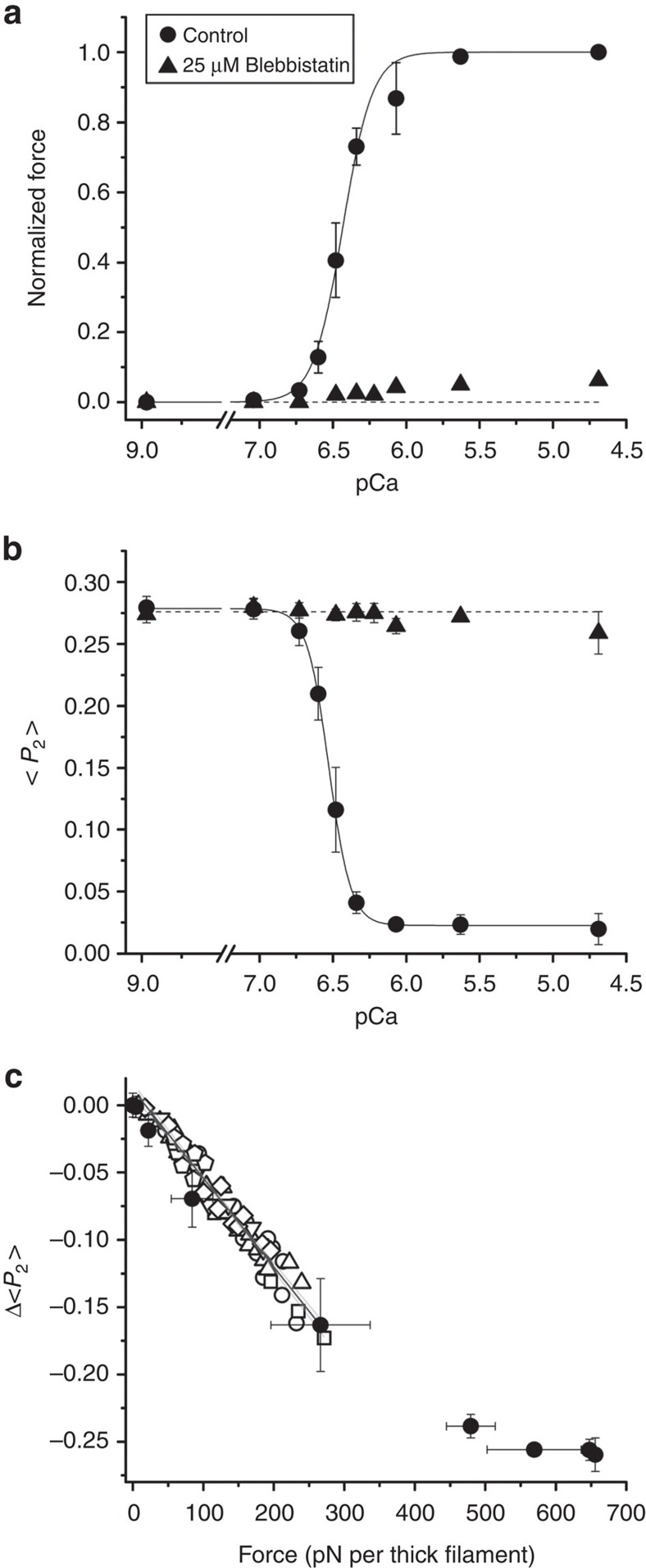
RLC orientation change during active isometric contraction. (**a**) Steady force at different pCa normalized by the maximum force at pCa 4.7 (267±10 kPa; mean±s.e., *n*=5 fibres) in the presence (triangles; mean±s.e., *n*=5 fibres; error bars are smaller than symbol size) and absence (circles; mean±s.e., *n*=5 fibres) of 25 μM Blebbistatin. Sarcomere length 2.40 μm, 25 °C, 5% Dextran. (**b**) <*P*_2_> for the E-helix probe in the same experiments. (**c**) Force-dependence of Δ<*P*_2_> during active contraction (filled circles, from **a**,**b**) compared with that for the end of phase 2 after force steps in the presence of 25 μM Blebbistatin (data from Fig. 4b).

To compare the changes in RLC orientation induced by passive and active force, we measured the order parameters <*P*_2_> and <*P*_4_> for the E-helix probe during active isometric contraction at different [Ca^2+^] at 25 °C in the presence of 5% Dextran but in the absence of Blebbistatin (Fig. 5 and Supplementary Fig. 10). The dependence of active isometric force on pCa (Fig. 5a, circles) was well described by the Hill equation (continuous line) with pCa_50_ 6.43±0.01 and Hill coefficient *n*_H_ 4.31±0.55 (mean±s.e., *n*=5 fibres). <*P*_2_> in the same fibres (Fig. 5b, circles) decreased with increasing [Ca^2+^], and these data were also well fitted by the Hill equation (continuous line) with pCa_50_ 6.53±0.01, *n*_H_ 5.80±0.17. Both the calcium sensitivity (pCa_50_) and co-operativity (*n*_H_) were significantly higher for <*P*_2_> than for force (paired two-tailed Student's *t*-test; pCa_50_ *P* value=0.002; *n*_H_ *P* value=0.04; *n*=5 fibres). The change in <*P*_2_> on activation, Δ<*P*_2_> (Fig. 5c and Supplementary Fig. 10c, filled circles) was approximately linearly related to active force for forces up to about 300 pN per thick filament.

When active force was almost completely inhibited by the addition of 25 μM Blebbistatin (Fig. 5a, triangles), <*P*_2_> for the E-helix probe became almost independent of [Ca^2+^] in these experiments (Fig. 5b, triangles), consistent with the results presented earlier (Figs 3 and 4). However, the relationship between <*P*_2_> for the E-helix probe and passive thick filament stress in the presence of Blebbistatin (Fig. 5c, open symbols) superimposes on that for active contraction in the absence of Blebbistatin (Fig. 5c and Supplementary Fig. 10c, filled circles) up to 300 pN per thick filament, indicating that passive and active stress have the same effect on RLC orientation in this range.

## Discussion

The results presented above show that the orientation of the RLC C-lobe region of myosin is sensitive to the passive stress induced by stretching a relaxed muscle fibre. From the perspective of established molecular paradigms of muscle contraction and its regulation, this is an unexpected finding. In relaxed muscle the intracellular calcium concentration is very low, the thin filaments are OFF, and the myosin motors lie on the surface of the thick filaments; a stretch in these conditions leads to relative sliding of the thick and thin filaments without interaction of the myosin motors with actin. The passive stress in these conditions is largely borne by titin links between the tips of the thick filaments and the ends of the sarcomere, in series with the thick filaments themselves, and the present results therefore show that the orientation of the RLC region of the myosin motors is sensitive to the stress in the thick filament.

**Figure 6:**
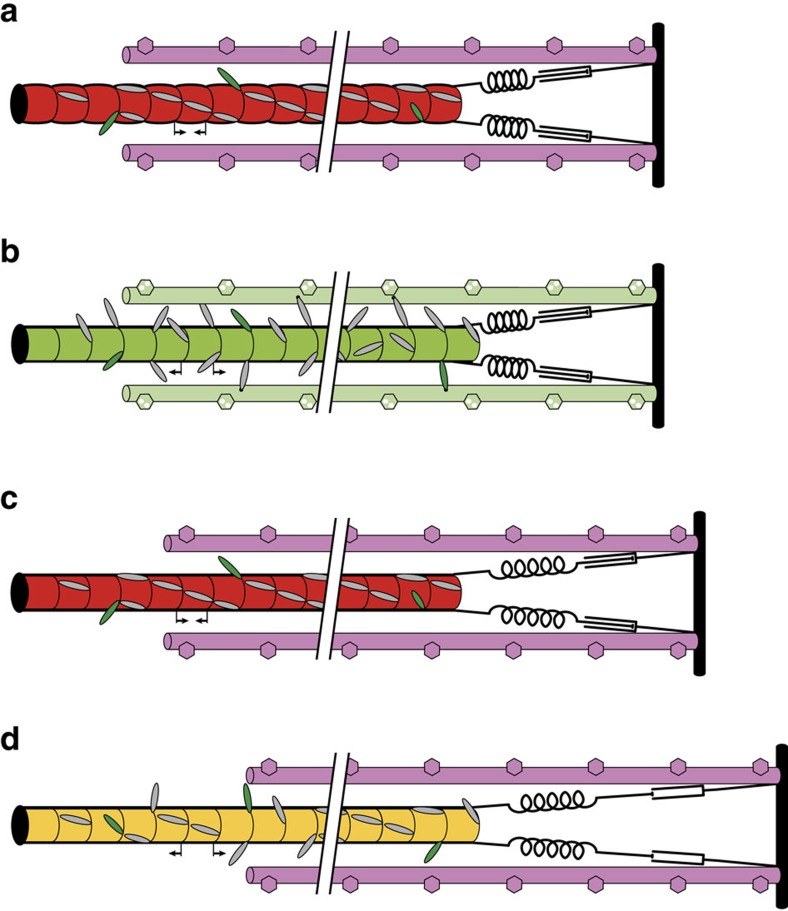
Thick filament stress controls the conformation of the myosin motors in skeletal muscle. The thick filament (red/yellow/green) is shown connected to the Z disk (vertical black bar) by visco-elastic titin links (black spring and dashpot) and partly overlaps with thin filaments (pink, light green). (**a**) In the absence of stress, the thick filament is OFF (red), with a short axial periodicity (inward pointing arrows) and helical tracks of myosin motors (grey ellipses). A few myosin motors are more perpendicular (constitutively ON; green). (**b**) Active contraction at high external load; both the thick filament (green) and thin filament (light green) are ON; the axial periodicity of the thick filament has increased (outward pointing arrows). (**c**) Elastic (phase 1) response to a force step in relaxing conditions; titin is elastically strained and passive force is transmitted to the thick filament which remains OFF. (**d**) Visco-elastic lengthening of titin in phase 2 is accompanied by the transition to a partially ON (yellow) thick filament with more myosin motors in perpendicular orientations.

This conclusion is consistent with the recent demonstration that the conformation of the myosin motors in actively contracting muscle is sensitive to the force borne by the thick filament, and the deduction from those results that the transition between structural and functional OFF and ON states of the thick filament is controlled by filament stress^14^. In the OFF or super-relaxed^9^,^10^ state of the thick filament (Fig. 6a), the majority of the myosin motors (light grey ellipses) lie in helical tracks on the surface of the thick filaments (red), but a small number of constitutively ON motors (green) are outside these helical tracks and available for interaction with the thin filaments (pink). When the thin filaments are activated by calcium, these constitutively ON motors are sufficient to drive muscle shortening at maximum velocity against a low external load^14^. When the external load is high in calcium-activated muscle (Fig. 6b), active force generation by the constitutively ON motors generates thick filament stress, triggering the transition to the ON state of the thick filament (green), characterized by a slightly (1.6%) longer filament backbone periodicity and the release of the majority of the myosin motors from their helical packing^8^,^14^, making them available for actin interaction and force generation against the high load.

The present results show that this thick filament mechano-sensing mechanism also operates in relaxed muscle, when the thin filaments are OFF (Fig. 6c,d, pink), and at low levels of RLC phosphorylation, when most of the myosin heads have parallel orientations with respect to the filament axis. In these conditions, filament stress is generated not by the constitutively ON motors, but by extension of the titin link (black) between the tip of the thick filaments and the end of the sarcomere, shown here schematically as an elastic spring in series with a dashpot, representing the viscoelastic response to a force step. Imposition of a passive fibre stress of 80 kPa (∼1/3 of the maximum calcium-activated force), equivalent to 200 pN per thick filament, induced a tilting of ∼15% of the RLC domains from the parallel RX2 and RX3 orientations that are characteristic of the OFF or super-relaxed state (Fig. 1b) to the more perpendicular RX1 orientation characteristic of the ON state. Thus the present results indicate that passive stress can partially switch ON the thick filament (Fig. 6d, yellow) triggering the release of the myosin heads from the OFF state. Consistent with this interpretation, pioneering X-ray studies by Haselgrove^29^ and more recent X-ray experiments on stretch of resting muscle^30^,^31^,^32^ demonstrated an increase in the spacing of the M6 reflection associated with the axial periodicity of the thick filament backbone and a decrease in the intensity of the first myosin layer line associated with the helical tracks of myosin motors in the OFF state. RLC phosphorylation also promotes the ON structure of the thick filaments in relaxed skeletal muscle^33^ and in cardiac muscle^34^.

The present results also show that the orientation of the RLC domain does not change during the elastic response to a force pulse (phase 1; Figs 2, 3, 4), but only during the next few milliseconds (phase 2). The change in the structure of the thick filament in response to stress is therefore not an instantaneous elasticity, and the thick filament is still OFF immediately after the force step (Fig. 6c). The elastic extension of the thick filament during the force step is much smaller than the 1.6% extension associated with the full OFF/ON transition; the elastic strain in the thick filament corresponding to the full isometric force of ca 180 kPa in intact frog muscle^35^ is only about 0.16%. We conclude that the elastic strain in the thick filament associated with extension of the titin links (Fig. 6c) triggers a structural transition in the thick filament (Fig. 6d) that has a rate constant of 100–500 s^−1^ depending on the force on the filament (Fig. 4c). The molecular structural basis of this transition is not fully understood, but it is linked to the change in the packing of the myosin tails in the thick filament backbone responsible for the 1.6% increase in filament periodicity, and the loss of the interaction between the myosin motors and other thick filament components that stabilize the helical tracks of motors in the OFF state. These interacting partners may include other regions of the same myosin molecule, other myosins, titin and myosin binding protein C.

Both the amplitude and the speed of the change in the orientation of the RLC C-lobe region of the myosin motors in response to a change in thick filament stress are independent of [Ca^2+^] (Figs 3 and 4), and the orientation of the E-helix probe has the same dependence on filament force during passive stretch and during steady activation at higher [Ca^2+^] up to forces of about 300 pN per filament (Fig. 5c). Thus the orientation of the RLC-C-lobe depends only on filament force and is independent of whether that force is active, generated by myosin motors, or passive, due to extension of the titin links. The orientation of the RLC C-lobe does change during the working stroke of the myosin motor at full calcium activation^36^,^37^, but at low levels of steady-state activation, up to forces of about 40% of the maximum observed at high [Ca^2+^], the structural signal associated with the working stroke is small compared with that associated with the OFF/ON transition in the thick filament.

Activation of the thick filament by mechanical stress (Fig. 6) implies a positive feedback loop in which, at a given calcium concentration, force generation by myosin motors increases thick filament stress, which in turn further increases thick filament activation. This positive co-operativity is likely to underlie the very steep [Ca^2+^]-dependence of the orientation change in the RLC C-lobe (Fig. 5b), which has a Hill coefficient of ∼6, much greater than that of force (∼4), or than that of thin filament activation (∼2 at 10 °C (ref. 38) and 20 °C (ref. 39)). The high co-operativity of thick filament activation might be mediated by intra-molecular interactions between heads in each myosin molecule or inter-molecular interactions between myosins or with other thick filament components. Thus mechano-sensing in the thick filament might contribute to rapid switch-like activation of muscle following electrical stimulation and the rise of intracellular [Ca^2+^] via a co-operative transmission along the thick filament. Since activation of the thin filament is much faster than force development^40^, it is likely that the rate of force development is limited by the slower structural transition, with a rate constant of 100–500 s^−1^ in the conditions studied here, that determines the rate of activation of the thick filament^14^. More generally, the present results provide strong support for the view that the regulation of contraction in skeletal muscle can be fully understood only by considering the roles of both thick and thin filaments in the mechanism, in contrast with conventional models.

## Methods

Four double-cysteine mutants of chicken skeletal RLC (D95C/V103C (E-helix), E131C/A138C (G-helix), K151C/T158C (H-helix) and T122C/K134C (linking F and G helices)) were obtained by site-directed mutagenesis and expressed in *Escherichia coli* BL21 (DE3) using a pET15b vector. The two native cysteines at position 125 and 154 were replaced by alanine to avoid non-specific labelling. Each pair of introduced cysteines was cross-linked with bifunctional sulfo-rhodamine (BSR, B10621, Invitrogen) to give 1:1 BSR:RLC conjugates that were purified by reverse-phase HPLC to >95% homogeneity. Specificity and stoichiometry of BSR labelling were determined by reverse-phase HPLC and mass spectrometry. BSR-RLCs were exchanged into demembranated fibres (length ∼5 mm) from the psoas muscle of adult (ca 18 weeks old) male New Zealand white rabbits. Muscle fibres were first transferred to rigor solution at ∼1 °C, then RLC-extracting solution (20 mM EDTA, 50 mM potassium propionate, 10 mM potassium phosphate buffer, pH 7.1) at 1 °C for 3′ and finally to RLC-extracting solution containing 0.5 mg ml^−1^ (∼25 μM) BSR-RLC at 19 °C for 30′. Fibres were washed in relaxing solution and then incubated at 10 °C for 20′ in relaxing solution containing 0.5 mg ml^−1^ of rabbit skeletal muscle troponin complex (Tn, Life Diagnostics, USA) and for a further 20′ in relaxing solution containing 0.5 mg ml^−1^ of wild-type chicken skeletal muscle troponin C (TnC). Fibres containing BSR-RLC were mounted in a custom-built setup to measure the polarized fluorescence of the BSR probes^40^ and their extremities were fixed using 5% glutaraldehyde in rigor solution and glued to the clips with shellac dissolved in ethanol. All the experiments were performed at 25 °C in the presence of 5% Dextran T-500 except where otherwise stated, conditions in which the physiological resting structure of the thick filament^7^ and the myofilament lattice spacing are recovered^41^. Passive and active force were normalized by the cross-sectional area of each fibre measured at 2.4 μm sarcomere length in the absence of Dextran, which was 3,043±466 μm^2^ (mean±s.d., *n*=15 fibres). Addition of 5% Dextran caused a ca 35% decrease in cross-sectional area. The force per thick filament was calculated dividing the normalized force by the thick filament density in the demembranated muscle fibres (0.407 × 10^15^ thick filaments per square metre)^30^,^42^.

In the calcium titration experiments, fibres exchanged with BSR-RLC E-probe were briefly (∼1–5 s) activated at 2.4 μm sarcomere length by transfer from pre-activating to activating solution at different pCa (=−log[Ca^2+^]) in the presence and in the absence of 25 μM Blebbistatin. Solutions with different pCa were obtained by mixing different proportions of activating (pCa 4.7) and relaxing (pCa 9.0) solutions^7^ and the final pCa was calculated using software kindly provided by Dr E. Homsher. Steady isometric force during maximal activation (pCa 4.7) at 25 °C was 267±10 kPa (mean±s.e., *n*=5 fibres), corresponding to 656±24 pN per thick filament, and <*P*_2_> and <*P*_4_> were recorded at the steady isometric force attained in each activation.

### Mechanical protocols

Single fibres were mounted between the levers of a strain-gauge force transducer and piezo-actuator (PZA12, Newport Corporation, USA). In the protocol used in Fig. 1, the sarcomere length was increased from 2.4 to 3.6 μm in relaxing solution by increasing the initial fibre length (*L*_0_) by 50% in steps of 0.05 *L*_0_ and 0.5 s duration applied at intervals of 10 s. A similar sequence of releases was applied to recover the initial fibre length.

In the force-clamp experiments a single muscle fibre containing RLC labelled with BSR on the E-helix was mounted between the levers of a capacitance force transducer and a loudspeaker motor^24^ to apply fast force pulses using force feedback. Before the force step was applied the sarcomere length was set to 2.8 μm to obtain a basal level of passive tension in the fibre that did not significantly alter the order parameters of the four BSR-RLC probes (Fig. 1). At this sarcomere length, the passive stiffness of the fibre is higher than that at 2.4 μm, and the effects of fibre inertia on the rapid force perturbations is minimized. Force was rapidly increased by stretching the fibre within 0.2 ms in fibre-length clamp mode. The force-clamp mode was imposed 0.2 ms after the start of the stretch, and after 50 ms force was returned to the pre-stretch value in 0.5 ms. The force decrease was not always accompanied by complete recovery of the initial sarcomere length (Figs 2, 3, 4) because of hysteresis in the passive force-sarcomere length relation (Supplementary Fig. 2).

### Fluorescence measurements

The polarized fluorescence intensities were used to calculate the second- and fourth-rank order parameters of the orientation distribution of the BSR dipole, <*P*_2_> and <*P*_4_>, respectively^18^,^40^ and the total fluorescence intensity *I* at 0.125 ms intervals during each mechanical protocol^18^. The sarcomere length change in the region of the fibre in which <*P*_2_> and <*P*_4_> were measured was estimated from the change in the fluorescence intensity ratio *I*_0_/*I*, in which *I*_0_ is the fluorescence intensity at the initial sarcomere length before the mechanical protocol (see Supplementary Methods and Supplementary Fig. 1).

To describe the *in situ* RLC orientation, we used the EG frame^7^ and the crystallographic structure of nucleotide-free myosin subfragment-1 from chicken skeletal muscle^19^. The orientation of the RLC domain is described by *β*, the angle between the E-helix (Fig. 1c, magenta) and the thick filament axis, and *γ*, the twist angle around the E-helix. *γ*=0 when the G-helix (Fig. 1c, orange) is in the plane defined by the E-helix and the thick filament axis, and *γ*>0 for a counter-clockwise rotation of the lobe viewed from the +end of the E-helix. The orientation of each BSR probe in this frame was combined with the corresponding measured order parameters using a maximum entropy algorithm to calculate the smoothest distribution of RLC orientations consistent with the data^20^. The two-dimensional contour map denoting the probability of each (*β*,*γ*) orientation (with 0°<*β*<180° and 0°<*γ*<360°) was projected onto the surface of a sphere using Origin software (OriginLab, Northampton, USA), in which *β* is the polar angle (latitude) and *γ* the azimuthal angle (longitude). When estimating the integral in spherical coordinates, the two-dimensional maximum entropy intensity distributions were multiplied by sin*β*. A binary mask was used to select the lobe containing populations RX2 and RX3. The boundary of the mask was defined by the contour line of the maximum entropy map corresponding to a probability value of 0.045 and by a line running along the local minimum in the intensity between the RX1 and RX2 lobes. Each map was multiplied by the mask and the integral over the selected region was divided by the total over the whole map to calculate the fraction of heads with RLC regions in the RX2 or RX3 peak^7^. Graphical representations of RLC orientations were generated using the PyMOL Molecular Graphic System (DeLano Scientific, LLC) and the RLC C-lobe structure from nucleotide-free chicken skeletal myosin^19^.

### Data availability

The data that support the findings of this study are available from the corresponding author upon request.

## Additional information

**How to cite this article:** Fusi, L. *et al*. Thick filament mechano-sensing is a calcium-independent regulatory mechanism in skeletal muscle. *Nat. Commun.* **7,** 13281 doi: 10.1038/ncomms13281 (2016).

**Publisher's note:** Springer Nature remains neutral with regard to jurisdictional claims in published maps and institutional affiliations.

## Supplementary Material

Supplementary Information Supplementary Figures 1-10, Supplementary Methods.
