# Supplementary material for: Thick filament mechano-sensing is a calcium-independent regulatory mechanism in skeletal muscle
Source: Nat Commun. 2016 Oct 31;7:13281. doi: 10.1038/ncomms13281 (PMC5095582; doi:10.1038/ncomms13281)
Supplement: Supplementary Information — Supplementary Figures 1-10, Supplementary Methods. [file ncomms13281-s1.pdf]

## Supplementary Figures

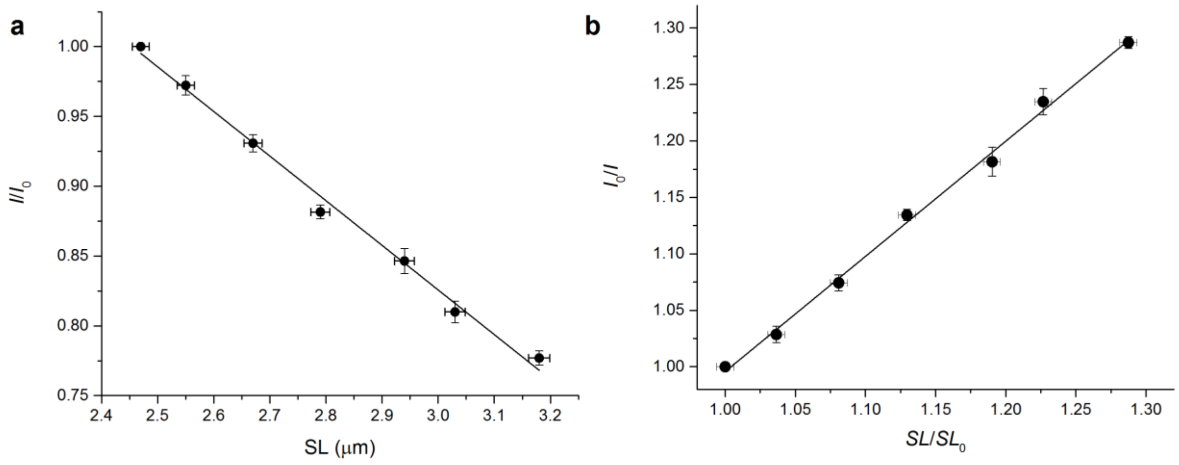


**Supplementary Figure 1. Sarcomere length-dependence of total fluorescence intensity in a relaxed muscle fibre containing BSR-RLC. a**) Fluorescence intensity (*I*) relative to the value at sarcomere length 2.45 µm ($I_0$), in a relaxed muscle fibre containing BSR-RLC (mean ± SD, n=3). Solid line: linear regression with slope -0.32 ± 0.01 µm$^{-1}$ and intercept 1.78 ± 0.03. **b**) Relation between fluorescence intensity ratio $I_0/I$ and sarcomere length relative to 2.45 µm ($SL_0$) (mean ± SD, n=3). Solid line: linear regression with slope 1.02 ± 0.03 and intercept -0.02 ± 0.03.

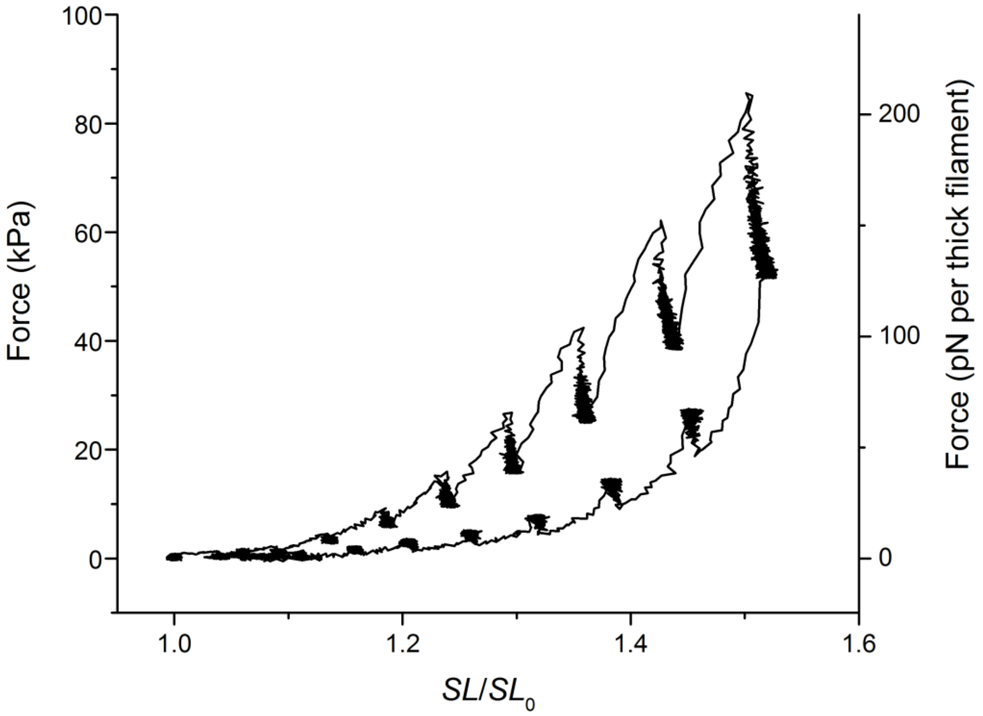


**Supplementary Figure 2. Sarcomere length-passive force relation in a relaxed muscle fibre.** Thick filament force (right axis), calculated from passive fibre force (left axis) as described in Methods, plotted against the sarcomere length relative to 2.45 µm ($SL_0$) during the staircase passive stretch-release protocol in Fig. 1.

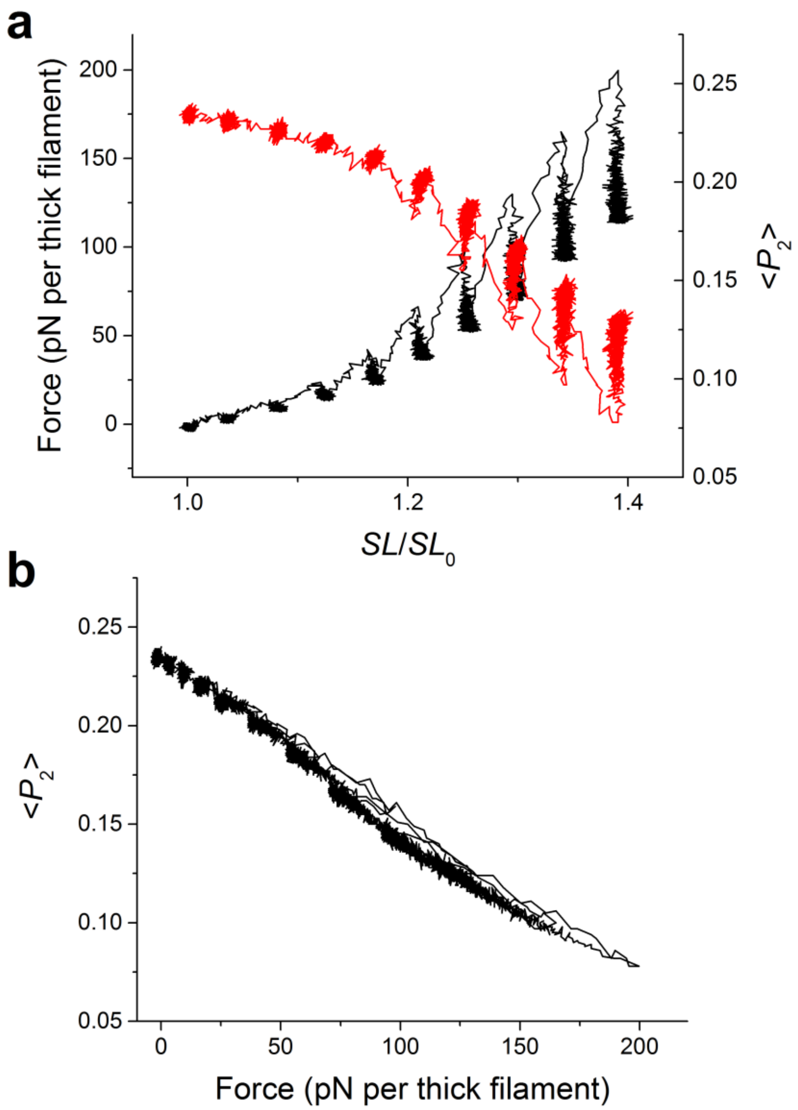


**Supplementary Figure 3. Dependence of the order parameter <$P_2$> for the E-helix probe on sarcomere length and thick filament force. a)** Plots of thick filament force (black) and <$P_2$> for the E-helix probe (red) against sarcomere length (*SL*) relative to the initial sarcomere length ($SL_0$ = 2.45 µm) during the staircase passive stretch protocol in Fig. 1. **b)** Plot of <$P_2$> against thick filament force shown in panel **a**.

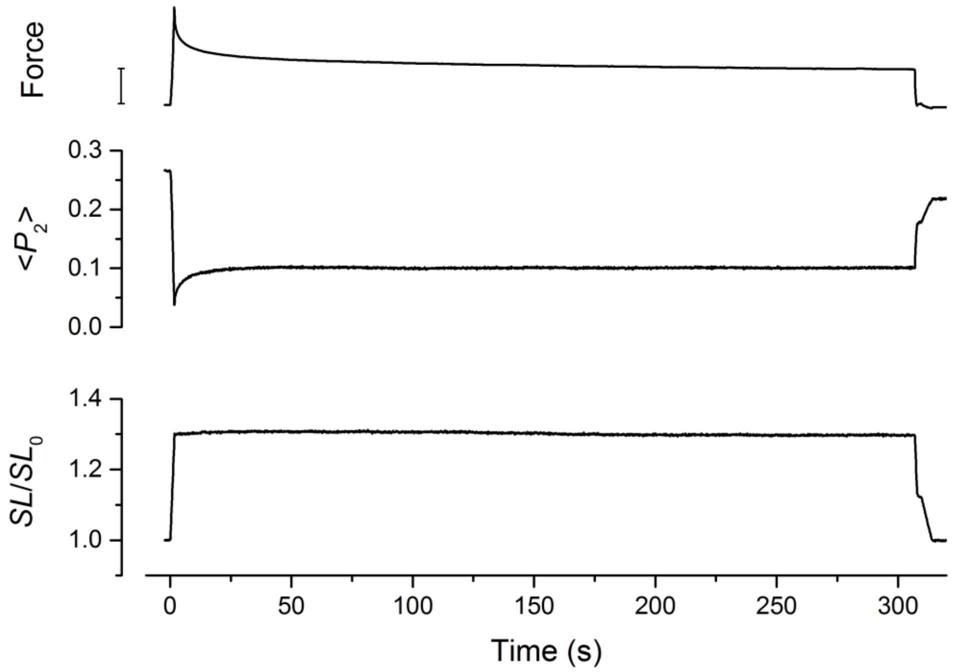


**Supplementary Figure 4. Order parameter <$P_2$> for the E-helix probe during a ramp stretch applied to relaxed muscle.** Upper panel: force (bar length = 120 pN per thick filament); middle panel: <$P_2$> for the E-helix probe; lower panel: sarcomere length (*SL*) relative to the initial sarcomere length ($SL_0$ = 2.45 µm). 25°C, 5% Dextran.

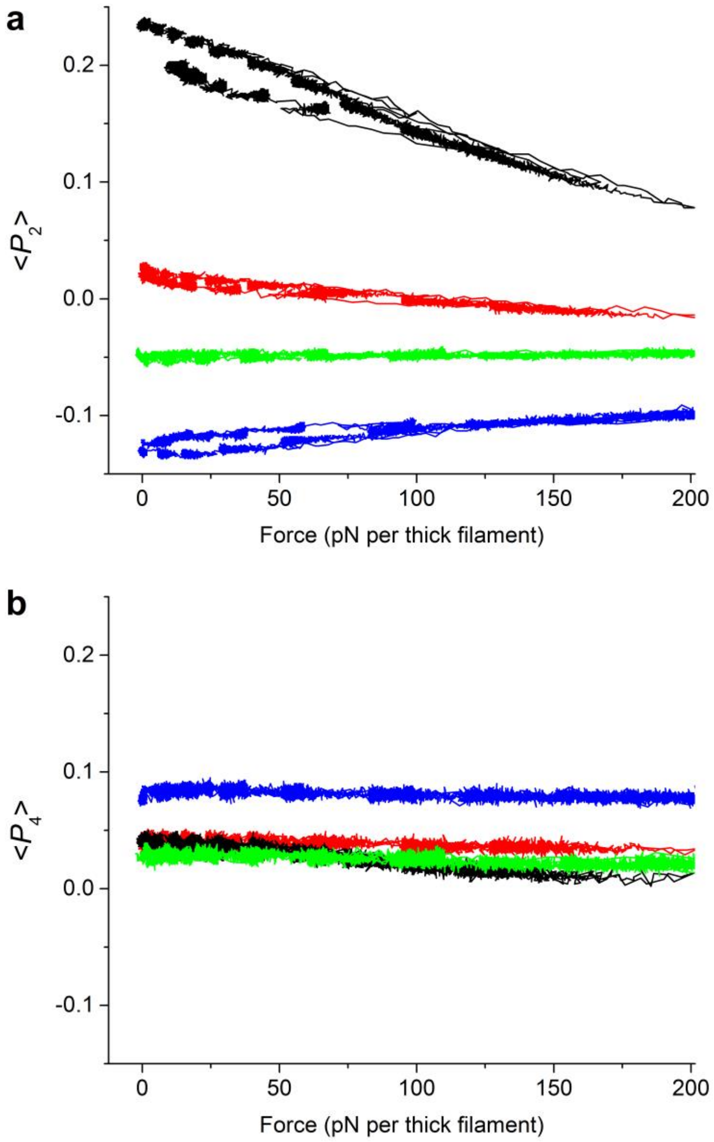


**Supplementary Figure 5. Dependence of the order parameters from four RLC C-lobe probes on thick filament force.** Plots of <$P_2$> (**a**) and <$P_4$> (**b**) for four RLC C-lobe probes (E, black; G, blue; FG, red; H, green) against thick filament force, recorded during the staircase passive stretch-release protocol applied to relaxed muscle fibres (Fig. 1).

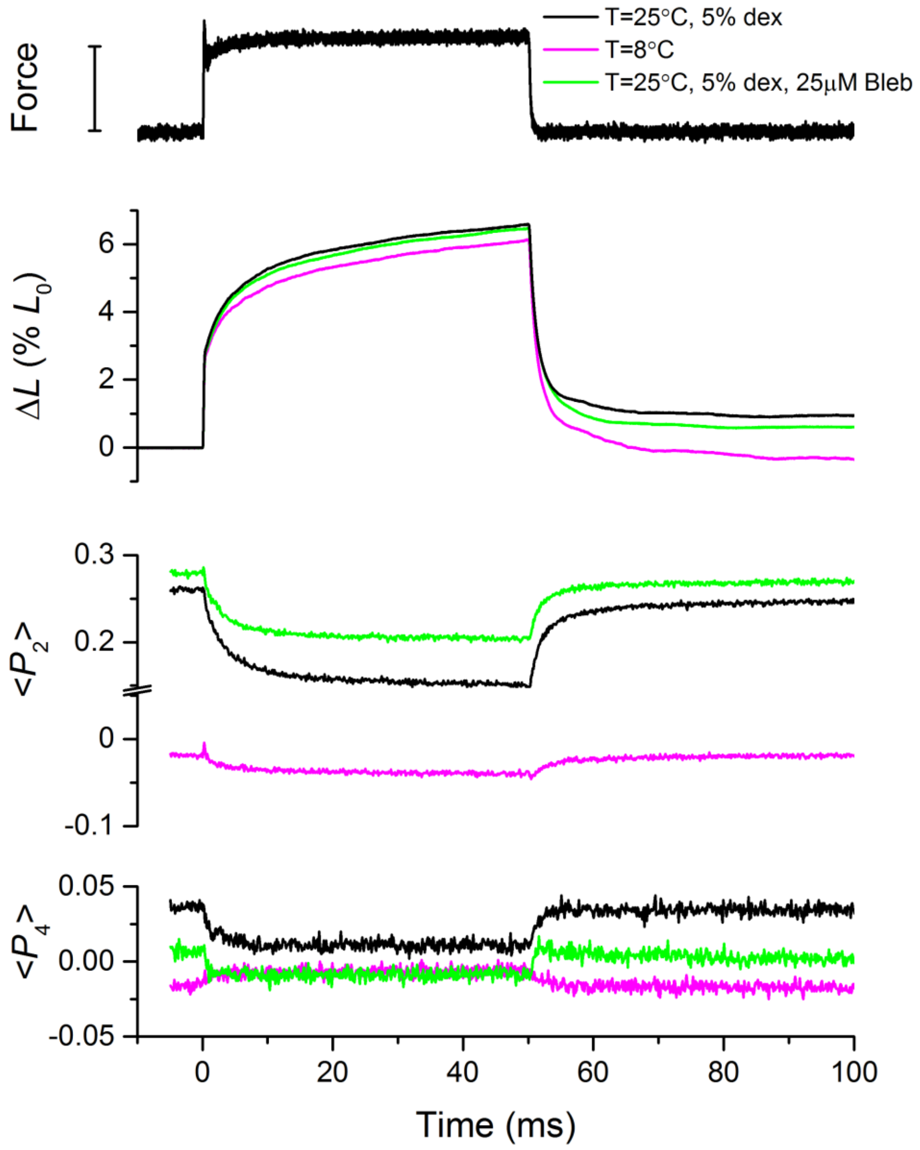


**Supplementary Figure 6. Order parameters for the E-helix probe during a force step applied to relaxed muscle.** Upper panel: force (bar length = 110 pN per thick filament); middle panel: fibre length change (Δ*L*) relative to initial fibre length ($L_0$); lower panels: <$P_2$> and <$P_4$> for the E-helix probe. Black: 25°C, 5% Dextran; green: 25°C, 5% Dextran, 25μM Blebbistatin; magenta: 8°C, no Dextran or Blebbistatin.

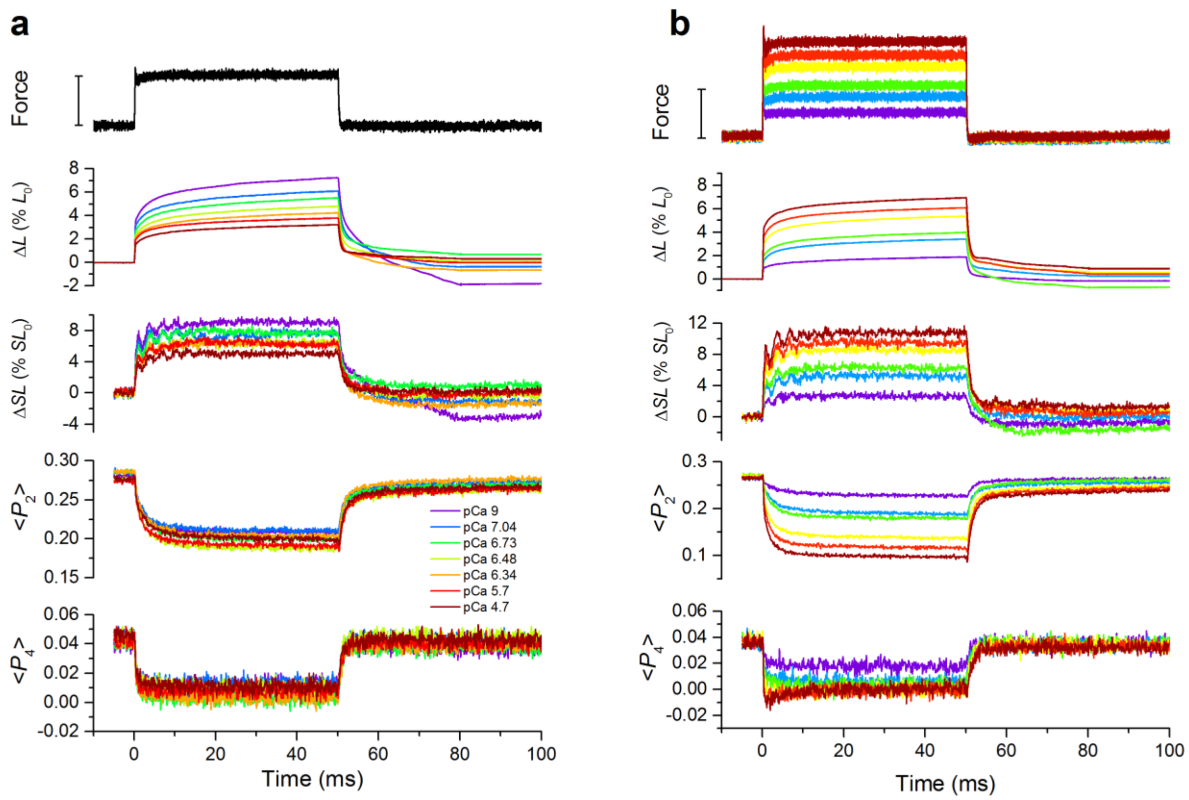


**Supplementary Figure 7. Calcium-dependence (a) and force-dependence (b) of the response of the order parameters for the E-helix probe to force steps.** Each panel shows force (bar length = 120 pN per thick filament), relative changes in fibre length (Δ$L$) and sarcomere length (Δ$SL$), and <$P_2$> and <$P_4$> transients for the E-helix probe. pCa = 5.86 in **b**. Initial sarcomere length 2.80 µm, 25°C, 5% Dextran, 25 µM Blebbistatin.

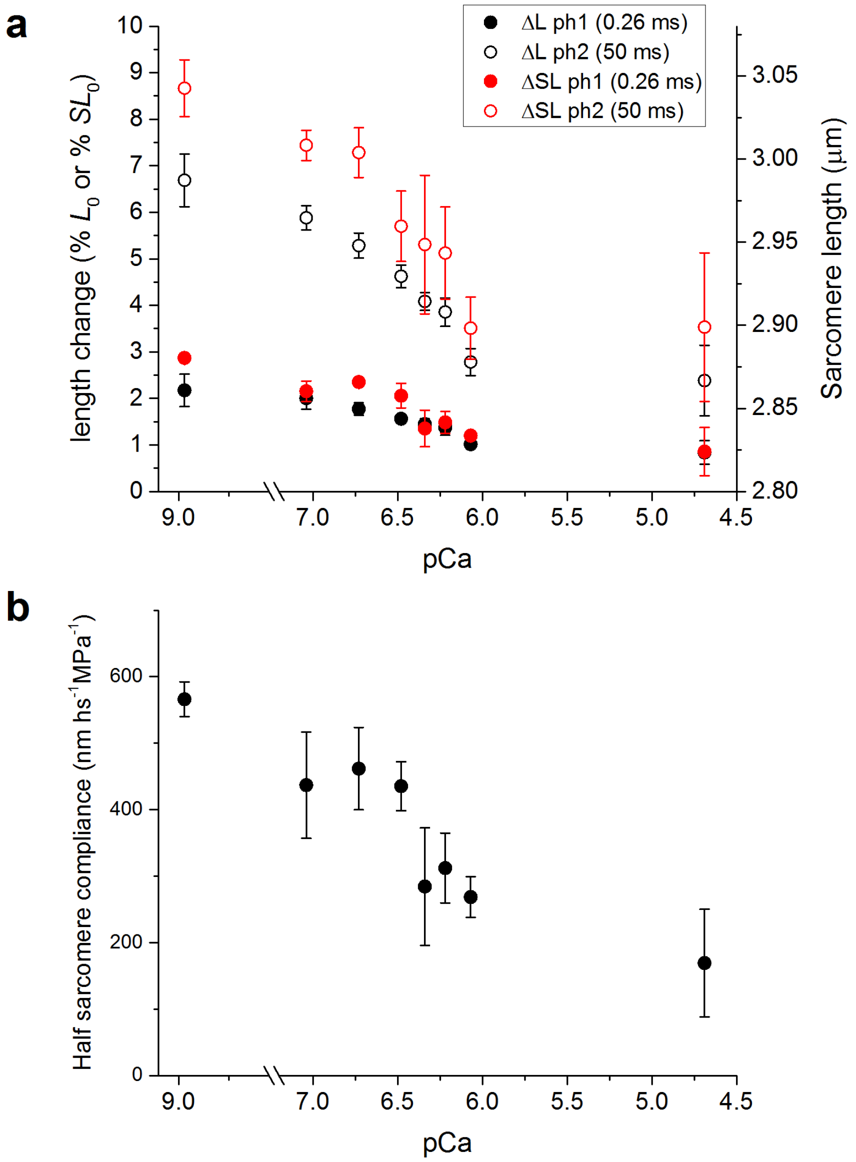


**Supplementary Figure 8. Calcium-dependent changes in the passive compliance of the half-sarcomere.** **a**) Calcium-dependence of fibre length change (black symbols as in Fig. 3**b**) and sarcomere length change (red) elicited by a 120-pN force pulse, measured at the end of phase 1 (filled circles) and phase 2 (open circles) (mean ± SD, n=3 fibres). **b**) Calcium-dependence of the passive compliance of the half-sarcomere estimated from the sarcomere length change in phase 1 in **a** divided by the amplitude of the force step in kPa. Initial sarcomere length 2.80 μm, 25°C, 5% Dextran, 25 μM Blebbistatin.

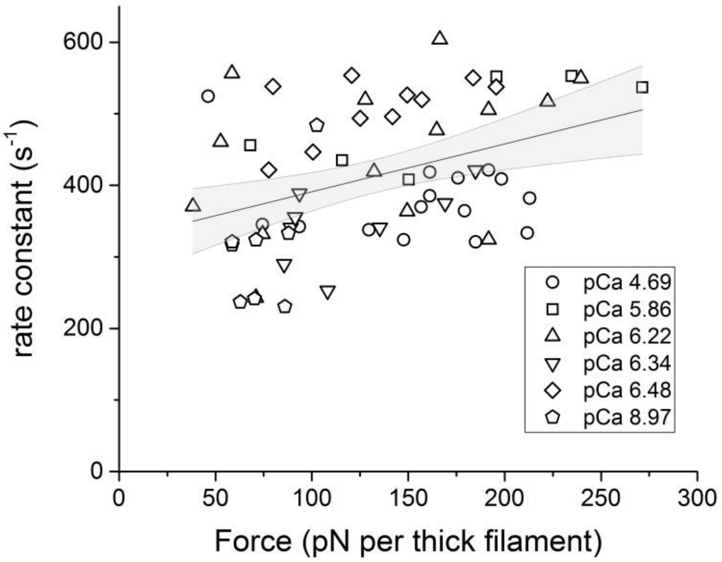


**Supplementary Figure 9. Rate constant of the <$P_2$> transient following the force decrease.** <$P_2$> transients for the E-helix probe in Fig. 4 for force steps from forces of up to 300 pN per thick filament to zero force (pooled data from 5 fibres), at different pCa. Solid line: linear regression with 95% confidence band in grey; slope = 0.67 ± 0.22 $s^{-1}pN^{-1}$ and intercept = 324 ± 30 $s^{-1}$.

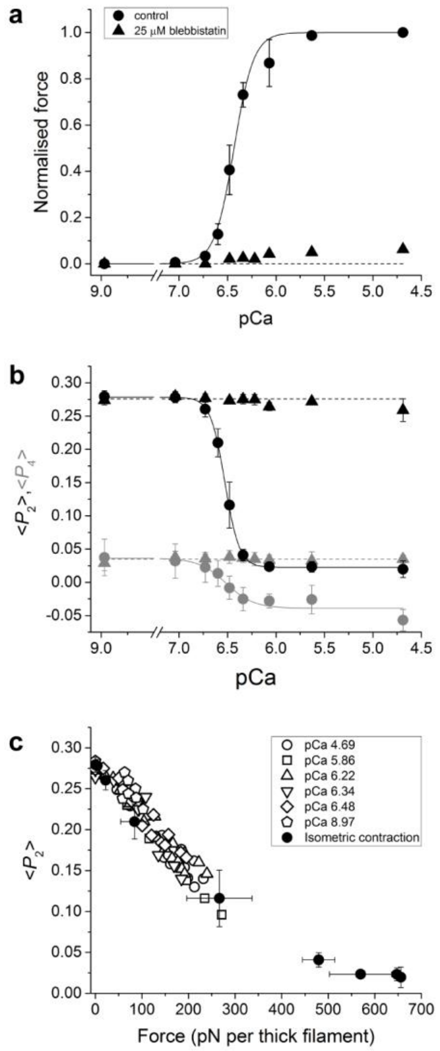


**Supplementary Figure 10. RLC orientation change during active isometric contraction. a**) Steady force at different pCa normalised by the maximum force at pCa 4.7 (267 ± 10 kPa; mean ± SE, n=5 fibres) in the presence (triangles; mean ± SE, n=5 fibres; error bars are smaller than symbol size) and absence (circles; mean ± SE, n=5 fibres) of 25 µM Blebbistatin. Sarcomere length 2.40 µm, 25°C, 5% Dextran. **b**) $<P_2>$ and $<P_4>$ for the E-helix probe in the same experiments. $<P_2>$ and $<P_4>$ data were fitted with the Hill equation with $pCa_{50}$ = 6.53 ± 0.01, $n_H$ = 5.80 ± 0.17 and $pCa_{50}$ = 6.51 ± 0.08, $n_H$ = 3.06 ± 1.45, respectively. **c**) Force-dependence of $<P_2>$ during active contraction (filled circles, from panels **a** and **b**) compared with that for the end of phase 2 after force steps in the presence of 25 µM Blebbistatin.

## Supplementary Methods

### Fluorescence intensity-based measurement of sarcomere length changes

Muscle fibres containing a BSR-RLC were mounted in relaxing solution between the levers of a force transducer and a motor. A short segment of the fibre was illuminated by a continuous diode-pumped solid-state laser in power-locked mode ($\lambda$=532 nm laser, Elforlight, LTD, UK) with a beam diameter of 560 µm (FWHM). The power on the sample was attenuated to ~0.5mW and under these conditions photobleaching produced an intensity decrease of only ~1% during a continuous 3-min exposure. Therefore changes in the total fluorescence intensity from BSR-RLCs are mainly associated with changes in the number of probes, and hence in the number of sarcomeres, illuminated by the laser beam. In order to determine the relation between total fluorescence intensity and sarcomere length we stretched fibres in relaxing solution and measured the total fluorescence intensity ($I$) and the sarcomere length ($SL$) using an optical microscope (40x immersion objective + 25x eyepiece) at three different points along the fibre length. Increasing the sarcomere length from 2.45 to 3.20 µm induced a linear decrease in total fluorescence intensity normalised to the initial value at 2.45 µm sarcomere length ($I/I_0$; Supplementary Fig. 1a). Changes in the fluorescence intensity ratio $I_0/I$ at longer fibre lengths were well correlated with the relative change in sarcomere length ($SL/SL_0$) (Supplementary Fig. 1b), indicating that $I_0/I$ gives a reliable measure of relative sarcomere length in these conditions.
